# Animal exposure, sensitization, and allergic symptoms in first-year veterinary medicine students 

**DOI:** 10.5414/ALX02449E

**Published:** 2024-03-21

**Authors:** Eva Zahradnik, Christoph Nöllenheidt, Ingrid Sander, Alexandra Beine, Martin Lehnert, Frank Hoffmeyer, Monika Raulf

**Affiliations:** Institute for Prevention and Occupational Medicine of the German Social Accident Insurance, Institute of the Ruhr-Universität Bochum (IPA), Bochum, Germany

**Keywords:** allergy, sensitization, animal exposure, veterinary medicine, cat, dog, horse

## Abstract

The AllergoVet study longitudinally examines the influence of animal exposure on the development of sensitization and allergic diseases among veterinary medicine students. In this group, contact to animals usually existed long before the study began. Therefore, the aim of this analysis was to investigate lifelong animal species-specific exposure and the prevalence of sensitizations and allergic symptoms already existing before the start of the study. Questionnaire data, including exposure history, were summarized to determine the duration and intensity of animal-related exposure as well as the prevalence of allergic symptoms to animals. Serologically, specific IgE was determined against ubiquitous inhalant allergens (atopy screen sx1) and against animal allergens using ImmunoCAP. The association between animal-specific sensitization, allergic symptoms, and exposure was analyzed using Fisher’s exact test or Cochran-Armitage trend test. All study participants (n = 313) had previous contact with animals, with dogs mentioned most frequently (91.1%) followed by cats (89.5%) and horses (72.2%). Sensitization to ubiquitous allergens (positive sx1 value) was detected in 38.4% of subjects. Approximately 11%, 7%, and 5% were sensitized to cats, dogs, and horses, respectively. Only a small proportion of these sensitizations were associated with self-reported symptoms (41% for cat, 9% for dog, and 13% for horse). While no significant association between animal-specific exposure and sensitization was found for cats and horses, a clear trend emerged for dogs. With increasing duration of exposure to dogs, the number of dog-specific sensitizations decreased significantly (p = 0.0069). Furthermore, a decreasing trend in sx1 sensitization was noted with increasing cat (p = 0.0288) and dog (p = 0.0107) exposure. None of the subjects who grew up on a farm (n = 40) had any sensitization to animals. The sensitization prevalence determined among first-year students in veterinary medicine roughly corresponds to that in the general population. Most animal sensitizations were not clinically relevant. In this collective, a protective effect of increasing exposure to animals in childhood and adolescence was found on sensitization, which was particularly pronounced during contact with dogs.

## Introduction 

Besides pollen and mites, furry mammals kept as companion, laboratory or farm animals are among the most common sources of allergens, causing development of sensitization and allergic diseases. The prevalence of sensitization to hair and dander from various animals appears to be increasing worldwide in the general population [1, 2, 3]. Animal allergy may also occur in various veterinary professions where workers are heavily and unavoidably exposed to animal allergens during most of their working time [4]. In any case, it is very likely that these individuals have contact with animals before and outside their respective occupations. The comprehensive review on occupational hazards in veterinarians showed a high prevalence of various allergic diseases ranging up to 63%, with the highest prevalence reported for respiratory symptoms. Rhinitis was the most frequent one, followed by cough/chest tightness, wheezing and airways obstruction [5]. The most recent study of female veterinary assistants even found a prevalence of 84% for rhinitis, 48% for lower airway symptoms such as cough, phlegm, and shortness of breath, and 11% for physician-diagnosed asthma [6]. However, it should be taken into account that a certain proportion of the allergic symptoms that were reported in various studies were not exclusively work-related and/or caused by the occupation. For example, in the U.S. survey of occupational hazards in veterinary practices, 65% of respondents with asthma and 46% with allergies reported having had these conditions already before working in veterinary medicine. Nevertheless, several study participants reported that their existing asthma or allergy symptoms had worsened since working in veterinary medicine [7]. It should also be remembered that not all work-related respiratory symptoms are due to animals. Veterinarians are additionally exposed to diverse chemical agents (e.g., ammonia, anesthetics, pesticides, pharmaceuticals, detergents, and disinfectants) and irritative microbial components such as bacterial endotoxin and β-glucans from molds [8]. However, of all work-related respiratory symptoms among California veterinarians, nearly 75% were related to specific animals, with cats being the most common species, followed by dogs, horses, cattle, and rabbits [9]. 

Veterinarians can experience substantial and simultaneous exposure to animal allergens of various species before their professional life, e.g., during their education at the university. In this regard, Samadi et al. [10] conducted a cross-sectional study in Dutch students of veterinary medicine at three different phases of their training and different specialization. This study provided evidence of increased prevalence of allergic symptoms with increasing duration of veterinary training, especially among individuals who handled farm animals. Paradoxically, the frequency of sensitization to any allergen decreased during training. To improve the knowledge about the development of animal sensitization and the occurrence of allergic symptoms in veterinary students, the prospective “AllergoVet” study was initiated at a veterinary university in Germany and conducted in cooperation with the Accident Insurance Hesse. 

The main objective of the present work was to investigate the prevalence of allergic symptoms and sensitization to animal allergens in first-year veterinary students recruited between 2013 and 2016. Furthermore, associations with species-specific lifetime exposures, particularly to the most common animal species cat, dog, and horse, were investigated. 

## Materials and methods 

### Study design and participants 

This cross-sectional investigation is a part of the project “AllergoVet”, a longitudinal study that explored the allergen exposure, development of sensitization and occurrence of allergic symptoms in veterinary students during their 6-year training. The study was conducted in cooperation with the Faculty of Veterinary Medicine of the Justus Liebig University in Giessen, Germany. The study was approved by a local Ethics Committee of the Ruhr University Bochum (registration number 4810-13). The recruiting of participants began in 2013 and ended in 2016, with a total of 313 students participating. The response rate for this longitudinal study was ~ 36% [11]. All study participants provided written informed consent and received financial compensation each year for their voluntary participation. 

The initial examination took place in November each year (~ 1 month after the start of training) and included a comprehensive questionnaire to record previous illnesses and animal exposures, a blood sampling, a spirometric examination, measurement of nasal nitric oxide (NO) and fractional exhaled NO (FeNO), and photographs of the hands to document skin symptoms. The analysis of NO and FeNO in regard to qualitative and quantitative aspects of sensitizations was already published by Hoffmeyer et al. [12]. Here, the focus is on the evaluation of the questionnaire and the serological determination of the specific IgE sensitizations. 

### Questionnaire 

The questionnaire included questions on demographic characteristics, smoking status, family history of allergy, farm childhood, current and previous contact with diverse animals, allergic symptoms to animals, and pre-existing allergic disorders. A farm childhood was derived from the question “As a young child (under 6 years old), did you live on a farm with close contact to farm or stable animals?”. To estimate the duration and intensity of direct and species-specific exposure to animals, several questions from the questionnaire were considered including pet ownership, close contact to animals in childhood or youth, animal-related education before university, internships, part-time jobs, and horseback riding or vaulting. Within these categories, contact with animals less than once a month was not taken into account. Indirect exposure, e.g., through contact with other animal owners, was not queried. For each study participant, the months of close contact with animals were counted and related to their age in percent. Self-reported allergic symptoms to animals were based on the affirmative answer to the question “Have you developed allergic symptoms to animals during your lifetime?” with an indication of the animal species (multiple responses possible). None of the study participants stated that they had given up keeping pets because of symptoms. Allergic diseases were only considered if they had ever been diagnosed or confirmed by a physician. 

### Total and specific serum IgE 

Sera were stored at –20 °C until IgE analysis was performed. Total serum IgE and specific IgE to cat dander (e1), dog dander (e5), horse dander (e3), cow dander (e4), and the house dust mite (HDM) *Dermatophagoides pteronyssinus* (d1) was quantified using Phadia ImmunoCAP 250 (ThermoFisher Scientific, Uppsala, Sweden). Atopy status in this study was defined as a sum of IgE specific antibodies against ubiquitous airborne allergens and determined with the inhalation allergy screening tool (sx1). Specific IgE values ≥ 0.35 kU/L were considered positive. Sera positive for animal dander were further analyzed for IgE antibodies against the following species-specific single components: rFel d 1 (e94, cat uteroglobin), rFel d 2 (e220, cat serum albumin), rFel d 4 (e228, cat lipocalin), rFel d 7 (e228, cat lipocalin), rCan f 1 (e101, dog lipocalin), rCan f 2 (e102, dog lipocalin), nCan f 3 (e221, dog serum albumin), rCan f 4 (e229, dog lipocalin), rCan f 5 (e226, dog prostatic kallikrein), rCan f 6 (e 230, dog lipocalin), rEqu c 1 (e227, horse lipocalin), and rEqu c 4 (horse latherin, in-house production using Streptavidin ImmunoCAP Ro210). For single animal allergen components, ImmunoCAP results ≥ 0.10 kU/L were considered positive. 

### Statistical analysis 

In the descriptive analysis of the sociodemographic and clinical aspects of the study participants, the differences between males and females were examined using Fisher’s exact test. Fisher’s exact test also was used to examine if sensitization to animal allergens was significantly associated with occurrence of symptoms. In sensitized subjects, the difference of specific IgE levels and the proportion of specific IgE to total IgE between those who reported symptoms and those who had no symptoms was investigated using non-parametric Mann-Whitney test. Cochran-Armitage trend test was used to analyze the relationship between animal sensitization and animal lifetime exposure. For this purpose, exposure categories were formed by dividing the collective into quartiles (for cat and dog) or terciles (for horse). Statistical analyses were performed using GraphPad Prism version 9.5.1 and SAS version 9.4 with level of significance set at 0.05. All reported p-values are based on two-sided tests. 

## Results 

### Subject characteristics 

Table 1 presents various characteristics of the study population. The participants were predominantly women (84.3%). The median age was 20 years (IQR: 19 – 22 years). There were significantly more current smokers among men (33%) than among women (10%). Approximately 13% grew up on a farm with animals. 80 participants (25.6%) already had an animal-related education before starting the study of veterinary medicine. Most of them (77.5%) were veterinary assistants or animal caretakers. Approximately 40% of the study population reported a history of allergy (disease diagnosed by a physician) with no gender differences for any disorder. The most prevalent allergic disease was rhinitis (16.9%), followed by atopic dermatitis (14.1%) and asthma (10.5%). Allergic symptoms (without specifying the type) to specific animals were reported by 30 individuals (9.6%), with cats being the most common trigger (n=23). All subjects had previous contact to animals, with dogs being mentioned most frequently (91.1%). Significantly more women than men reported contact with horses and small mammals. More than one-third of the study participants (36.7%) kept at least one pet in their current apartment/residence. The most common pets were dogs, followed by cats, which were mostly preferred by women. 

### Serological parameters 

The results of the serological evaluation are presented in Table 2. The median concentration of the entire collective (n = 313) for total IgE was 40.2 kU/L, with 26.5% being above 100 kU/L. Atopy, with respect to sx1 level, was identified in 38.4% of subjects, with a significant difference between men and women (57.1 vs. 34.8%; p = 0.004). The overall prevalence of HDM sensitization was 24.3%, being also more frequent in males than females (34.7 vs. 22.3%), but without statistically significance. Animal-specific sensitization was much rarer compared to HDM sensitization. Sensitization to animals was most frequent for cats (10.9%), followed by dogs (7.0%), horses (5.1%), and cattle (2.6%). No gender differences were observed. Overall, 47 subjects (15%) were sensitized to at least 1 animal allergen tested. Of them, 14 (30%) were not sensitized to HDM, and only 2 (4%) were not positive for sx1. 

### Animal specific sensitization and symptoms 

**Figure 1.**
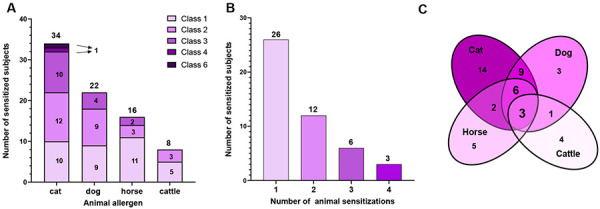
Sensitization profile of 47 individuals with specific IgE reactivity to animals. A) Distribution of CAP-classes. B) Number of animal sensitizations. C) Distribution of mono- and poly-sensitizations.

**Figure 2.**
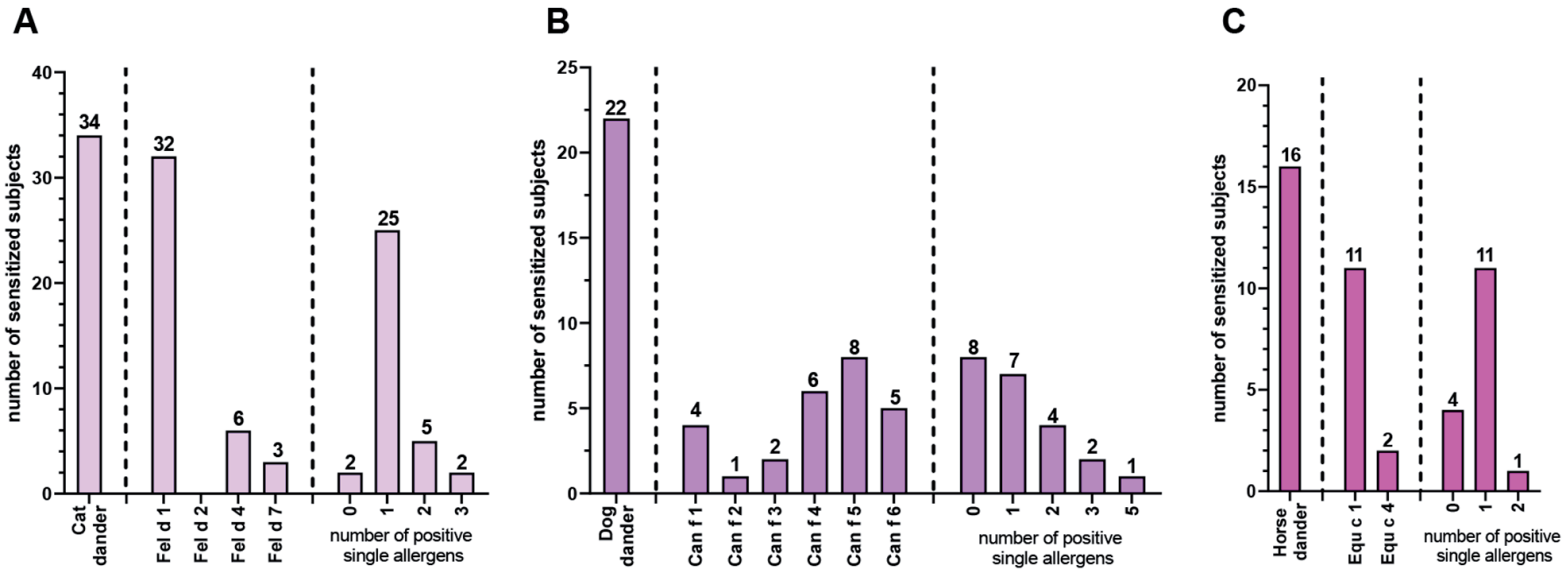
Distribution of single allergen components in subjects with sensitization to cat (A), dog (B), and horse (C).

Positive results for animal sensitizations were further classified according to the CAP classes (Figure 1A). Most of the sensitized subjects had low or moderate specific IgE levels (CAP class 1 – 3). Very high specific IgE levels (CAP class 4 and 6) were found only in 2 cat-sensitized individuals. Of the 47 subjects with animal sensitization, 55% were mono-sensitized, 26% double-sensitized, and 13% and 6% poly-sensitized to 3 or 4 animals, respectively (Figure 1B). Mono-sensitization was most common for cat and least common for dog, and dual sensitization to cat and dog was the most common species combination. The distribution of mono- and poly-sensitizations is depicted per Venn diagram (Figure 1C). Animal sensitizations were also analyzed with respect to available individual components (Figure 2). Of the 34 subjects showing sensitization to cat, almost all were positive to Fel d 1 (94%), and only few reacted to Fel d 4 (18%) and Fel d 7 (9%), respectively. In the sera of 2 participants sensitized to cat, no specific IgE to a single cat allergen was detectable. Can f 5 was the most prevalent positive component in subjects with dog sensitization (36%), followed by Can f 4 (27%), Can f 6 (23%), and Can f 1 (18%). One third of dog-positive subjects did not react to any single allergen, and another third was positive to only 1 component. Within the group sensitized to horse, 69% had elevated IgE levels to Equ c 1 and 12.5% to Equ c 4. Four individuals (25%) showed no reaction to any horse component. 

**Figure 3.**
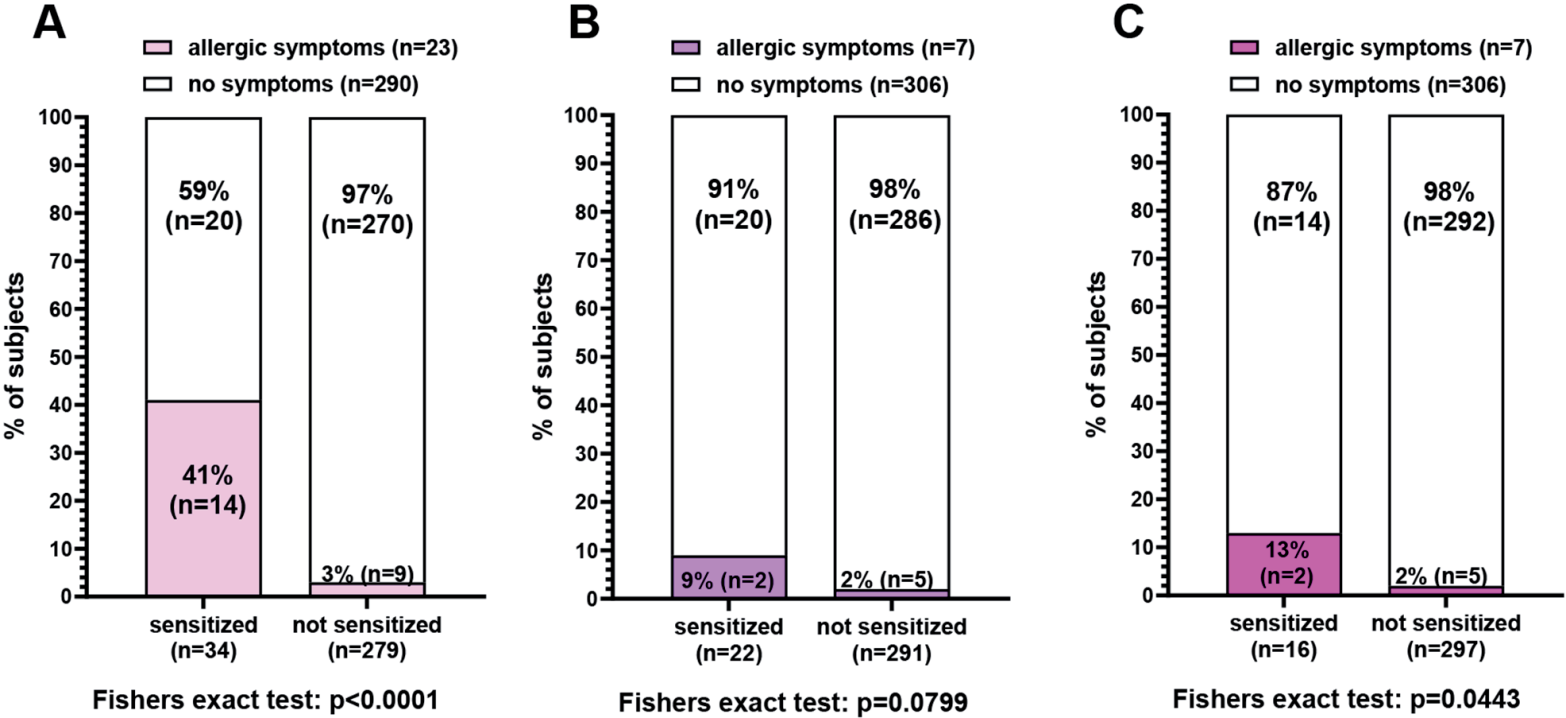
Relationship between sensitization and self-reported allergic symptoms to cats (A), dogs (B), and horses (C).

**Figure 4.**
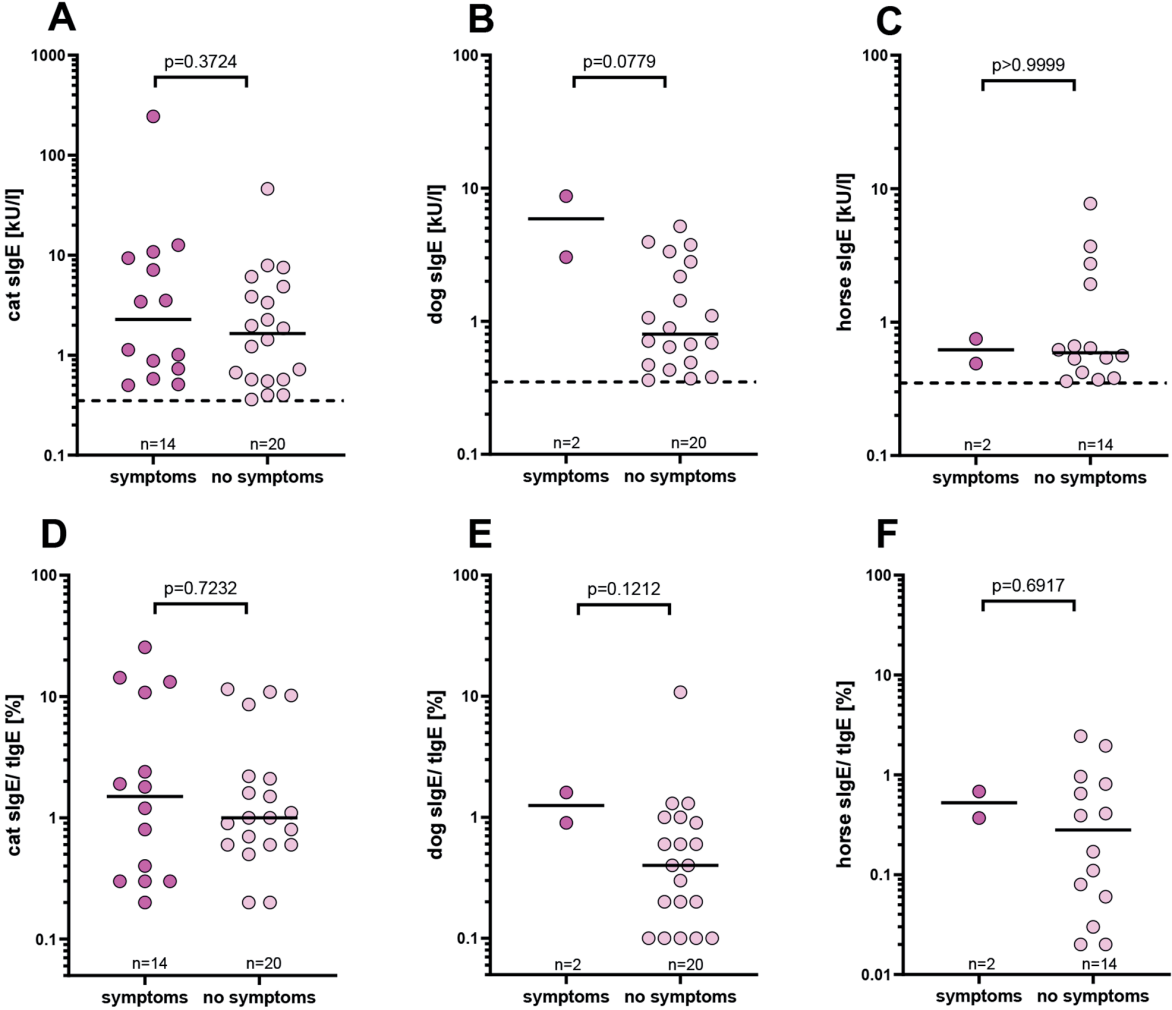
Specific IgE levels (A, B, C) and the proportion of specific IgE to total IgE (D, E, F) in subjects sensitized to cats (A and D), dogs (B and E), and horses (C and F). The vertical solid lines represent medians, and the dashed lines show the IgE cut-off value of 0.35 kU/L. Statistical significance was tested using the Mann-Whitney test.

To assess the clinical relevance of species-specific sensitization, subjects were asked about allergic symptoms to specific animals in a questionnaire. Symptoms to cats were reported by 23 individuals and to dogs and horses by 7 individuals each. Self-reported allergies correlated significantly with the presence of specific IgE to allergens from cats and horses, but not from dogs (Figure 3). However, the proportion of clinically relevant sensitizations was rather small (41% for cat, 9% for dog, and 13% for horse). The number of participants reporting allergic symptoms without a sensitization to the respective source was generally low (3% for cats and 2% for dogs and horses). The proportion of IgE reactivity to specific sources without allergic symptoms was very high (59% to cats, 91% to dogs, and 87% to horses). In the sensitized individuals, the specific IgE levels did not differ significantly between those who reported symptoms and those without symptoms. The same was true for the proportion of specific IgE to total IgE (Figure 4). 

### Relationship between animal sensitization and animal exposure 

**Figure 5.**
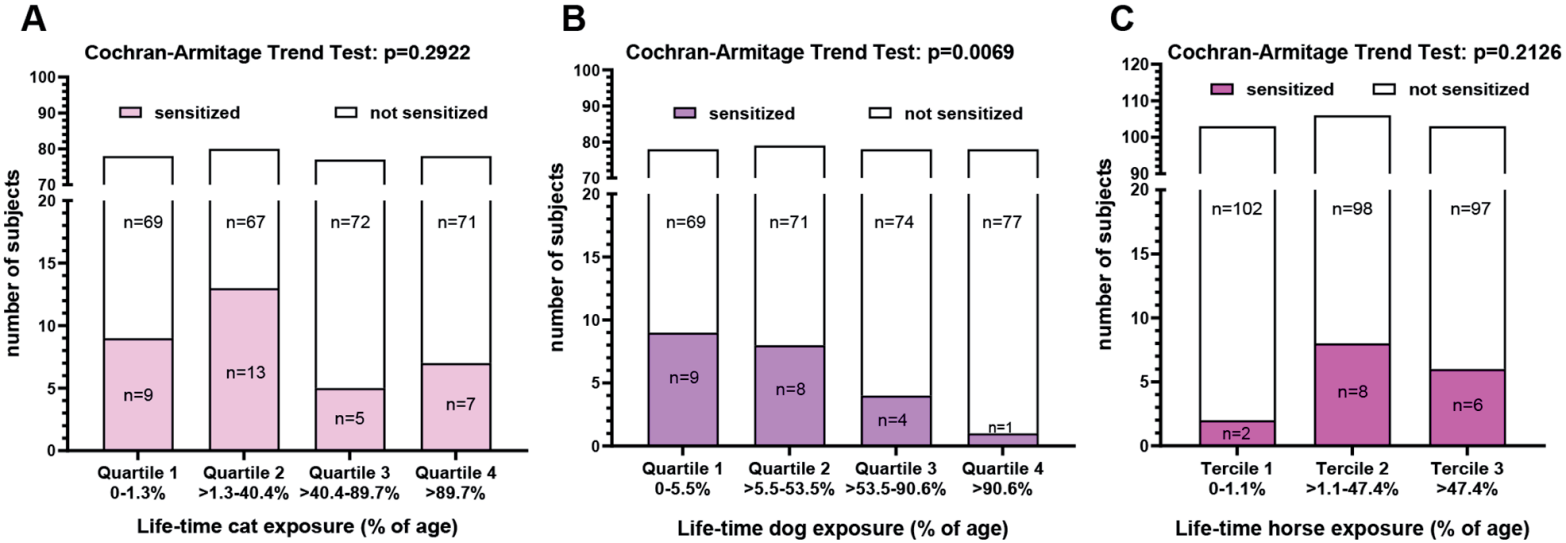
Relationship between sensitization and categories of life-time exposure to cats (A), dogs (B), and horses (C).

Furthermore, the species-specific relationship between animal sensitization and animal exposure was analyzed. For this purpose, the number of months of close contact with animals were added up for each study participant and related to their lifetime in percent. Many of the participants had intensive contact with dogs for more than half of their lives, and only a quarter had no or very rare contact. Intensive contact with cats was only slightly less frequent. One third had intensive contact with horses for almost half of their lives while another third had almost no contact with horses. Accordingly, the collective was divided into quartiles or terciles with regard to their animal contact and related to the number of sensitizations (Figure 5). While no association between exposure and sensitization was found for cat and horses, a clear trend was observed for dogs. With increasing dog exposure, the number of dog sensitizations decreased significantly (p = 0.0069). In addition, some significant effects were seen when analyzing the relationship between atopic status and animal exposure. A declining trend of sx1 sensitization was found with increasing cat (p = 0.0288) and dog exposure (p = 0.0107). Moreover, none of the subjects (n = 40) who grew up on a farm had any animal sensitization. 

## Discussion 

The prevalence of allergic sensitizations to animals depends on several factors such as the study group, predisposition to atopy, age, sex, and country. In Europe, the sensitization rate for cat varies from 16.8 to 49.3% in patient populations [13] and from 1.2 to 22.4% in the general population [14], being particularly high in Nordic countries and lower in Central/Western and Mediterranean countries It is also known from longitudinal studies that the frequency of animal sensitization increases during childhood and adolescence and peaks in young adulthood [15, 16, 17, 18]. In our study population of young adult veterinary students, ~ 11, 7, and 5% were sensitized to cats, dogs, and horses, respectively. These rates are about half lower than at the 24-year follow-up in the Swedish population-based birth cohort BAMSE (19.6% for cat, 16.9% for dog, and 9.8% for horse) [18] and rather comparable to the recent Austrian population-based study LEAD with a frequency of positive skin prick test to cat and dog in childhood/adolescence (11.4 and 6.7%) and in adulthood (13.7 and 11.4%), respectively [3]. The “German Health Interview and Examination Survey for Adults (DEGS)” has reported prevalence of sensitization to animal dander (cat, dog, horse) of 15.4% in the age group of 18 – 29 years [19] which is similar to our result of 13.7% (cat, dog, horse). In contrast to other studies [3, 15, 17, 18], we did not observe a significantly higher prevalence of sensitization to any animal allergen among males compared to females. A statistically significant difference between males and females (57.1 vs. 34.8%) was only found in positive IgE reactions to aeroallergens (sx1) which is, on the other hand; consistent with the studies mentioned above [3, 15, 17, 18]. 

Consistently with other studies [20, 21], about half (45%) of all subjects with animal sensitization reacted to more than one animal species, and mono-sensitization to cats was most common (41%). Poly-sensitization can either be based on independent sensitizations or on immunological cross-reactions of antibodies binding to related animal allergens (or combination of both). To date, there are only a few studies on the molecular background of such poly-sensitization and its clinical relevance. Allergy diagnosis using whole extracts cannot differentiate between primary sensitization and cross-reactivity. To clarify the primary allergen source, it is helpful to perform component-resolved diagnostics (CDR). In our study, the analysis of single allergens in animal-sensitized subjects was in line with the finding that the most prevalent components were Fel d 1 for cat, Can f 5 for dog, and Equ c 1 for horse [20, 21, 22, 23]. In contrast, the frequency of IgE sensitization to all other cat and dog components was much lower than in other studies using CDR, particularly for Can f 1 which is usually the second most common allergen in dog sensitized patients. No or only very few positive reactions were found for the albumins Fel d 2 and Can f 3. Therefore, we can exclude that the poly-sensitizations occurring in our study are caused by the cross-reactive serum albumins, which was suggested by other authors [24, 25]. Another possible reason for poly-sensitization is the cross-reactivity shown between the lipocalins Can f 1 and Fel d 7 [26] or Can f 6/Fel d 4 /Equ c 1 [27, 28]. Due to low the number of subjects with positive reactions to these lipocalins, we were not able to investigate this phenomenon in more detail for different combinations of double- and poly-sensitization. However, in subjects simultaneously sensitized to cat, dog, and horse, the number of recognized single allergens was higher than in mono- or double-sensitized subjects (data not shown). 

There is not enough evidence to predict whether sensitization to animals will result in clinical allergy or not [29]. It is well known that some proportions of sensitizations do not become clinically relevant. A large patient-based study, GA^2^LEN, reported that the percentage of clinically relevant cat and dog sensitizations across Europe was 74% and 60%, respectively [30]. In our study, the rates were much lower (41% for cat, 9% for dog, and 13% for horse) (Figure 3). Possible explanations for these differences would be: 1) young students compared to a patient population, 2) high proportion of low IgE levels (CAP class 1 – 3) in our study group, 3) interference of symptoms with other respiratory allergies (e.g., mites), and additionally 4) unconscious suppression of symptoms due to affection for animals. A limitation of our study is that allergic symptoms to animals were based on participants’ self-assessment, and we did not perform any challenge tests. On the other hand, a recent study using a nasal provocation test to examine the association between IgE sensitization to animal dander (cat and dog) and clinical symptoms concluded that although sensitization to animal dander identifies atopic individuals, its utility in predicting clinical relevance is low [31]. However, despite the low occurrence of symptoms, the association between sensitization and clinical relevance was statistically significant for cats and horses, but not for dogs. This may be attributed to the fact that Fel d 1 and Equ c 1 met the criterion for a major allergen with an IgE reactivity of 94% and 69% in all cat- and horse-sensitized subjects, respectively. No major allergen was found in dog-sensitized subjects. Moreover, ~ 1/3 of dog-sensitized subjects did not react with any single allergen, and another third reacted to only one. However, clinically relevant dog allergy is associated with multi-sensitization to dog allergen components [32]. Accordingly, the 2 subjects with symptoms to dogs in our study were sensitized to three individual allergens. 

The association between pet ownership and the development of allergic diseases and asthma has been extensively investigated in numerous cross-sectional and cohort studies predominantly in early childhood, but also among adolescents and adults. The results of these studies are inconsistent and partly contradictory, with increased risk of pet sensitization, no influence, or even protective effects [33, 34]. The heterogeneity of results might be explained by several factors such as differences in pet keeping (indoor/outdoor), pet avoidance behavior, and degree of pet exposure. According to several meta-analyses, there is ultimately no clear evidence that pet ownership in childhood is associated with an increased risk of allergy or asthma [35, 36]. Unfortunately, no such studies are available for horses. However, for pet-specific or allergic sensitization in general, the overall trend is that pet ownership is associated with lower risk, and this is seen more commonly for dogs than for cats. Moreover, according to recent studies, keeping pets in childhood appears to have a protective effect well into adulthood [37, 38]. In our study, we found a clear and significant protective effect of lifetime duration of dog exposure on specific sensitization to dogs as well as on sensitization to aeroallergens (sx1). For exposure to cats, the protective effect was found only for sensitization to the ubiquitous allergens detected with sx1, and no effects were found for exposure to horses. In contrast to most other studies that classified the animal exposure by current and/or previous pet ownership (yes/no), we conducted a dose-dependent analysis. Because of the comprehensive questionnaire, we were able to consider both the duration and intensity of contact with animals over the lifetime of the study participants and form exposure categories. To our knowledge, this has never been done this way before. Our results suggest that not only contact with animals in childhood but also prolonged and intense exposure to pets may be required to achieve the protective effect in allergic sensitization. Since many people do not start riding in early childhood, this is probably why there is no such trend with horses. A further explanation for the dose-dependent protective effect of keeping pets is provided by the study by Hasselmar et al. [39], according to which it is not the animal species but the number of pets that plays a much more important role. Considering the characteristics listed in Table 1, the veterinary students must also have had contact with multiple animals and multiple animal species during their lives. 

The exact mechanism of an allergy- or sensitization-protective effect is still unclear, but several possible explanations have been proposed. First, the keeping of pets leads to extremely high allergen concentrations from the respective species in the household and could therefore induce clinical tolerance to the allergen [40]. Second, pet ownership is strongly associated with high indoor levels of endotoxin [41, 42] which protect against allergies by inducing the production of A20 in lung epithelial cells [43]. Third, living with pets is related to alterations of the composition and diversity of the skin and gut microbiome [44, 45, 46], which provides crucial signals for the development and function of the immune system [47]. All these factors can cause a so-called “farm effect” known to protect against asthma and allergies, especially in children [48, 49]. Consistently, no one in our study who grew up on a farm with animals had any animal sensitization. Childhood on a farm was also protective against sensitization to allergens (OR = 0.6) in a Dutch study among veterinary students [10]. 

A strong limitation of our study is that we did not adjust our data for potential confounding factors. Several factors including family history of allergy, presence of siblings, socio-economic status, and housing factors should be considered in studies on pet exposure and allergic diseases or sensitizations [50]. On the other hand, although we included more than 300 participants in our study, the size of the special cohort and especially the number of sensitized subjects is too small to perform multivariable analyses. Furthermore, a previous analysis of our study population suggests a “selection bias””, as asthma patients among the students seemed particularly motivated to participate in the AllergoVet study due to their health situation. A pre-existing allergy might have increased the interest in participating in this prospective study [11]. 

## Conclusion 

The prevalence of sensitization to animal allergens determined among first year students in veterinary medicine roughly corresponds to that in the general population in Central Europe in this age group. However, most sensitizations were not associated with allergic symptoms, particularly those to dogs and horses. Although clinically relevant sensitization was present in some individuals, these subjects did not avoid choosing training that required intensive contact to animals. In the animal-friendly population studied here, a protective effect against sensitization caused by prolonged animal exposure over the course of life was demonstrated, which was particularly pronounced in contact with dogs. 

## Acknowledgment 

The authors thank the dean and the administration of the Faculty of Veterinary Medicine of the Justus Liebig University Giessen for making it possible to conduct the study. 

## Funding 

This study was financially supported by the German Social Accident Insurance (DGUV) and in part by the Accident Insurance Hesse (Unfallkasse Hessen). The funders had no role in study design, data collection and analysis, decision to publish, or preparation of the manuscript. 

## Conflict of interest 

All authors declare that no conflict of interest exists. 

**Table 1.** Descriptive characteristics of study participants.

**Characteristic**	**Total ** **(n = 313)**	**Females ** **(n = 264)**	**Males ** **(n = 49)**	p-value*
Age, median (range)	20 (17-42)	20 (17-42)	20 (18-33)	
Current smoker, n (%)	43 (13.7)	**27 (10.2)**	**16 (32.7)**	**0.0002**
Childhood on farm with animals, n (%)	40 (12.8)	34 (12.9)	6 (12.2)	> 0.9999
Animal-related education before the veterinary study#, n (%)	80 (25.6)	64 (24.2)	16 (32.7)	0.2166
Allergic disease** (any),	124 (39.6)	106 (40.2)	18 (36.7)	0.7510
Asthma, n (%)	33 (10.5)	27 (10.2)	6 (12.2)	0.6187
Rhinitis, n (%)	53 (16.9)	44 (16.7)	9 (18.4)	0.8356
Neurodermatitis, n (%)	44 (14.1)	40 (15.2)	4 (8.2)	0.2639
Contact dermatitis, n (%)	10 (3.2)	9 (3.4)	1 (2.0)	> 0.9999
Urticaria, n (%)	16 (5.1)	14 (5.3)	2 (4.1)	> 0.9999
Food allergy, n (%)	30 (9.6)	28 (10.6)	2 (4.1)	0.1929
Insect allergy, n (%)	9 (2.9)	8 (3.0)	1 (2.0)	> 0.9999
Allergic symptoms to animals*** (any)	30 (9.6)	24 (9.1)	6 (12.2)	0.4391
Cat, n (%)	23 (7.3)	20 (7.6)	3 (6.1)	> 0.9999
Dog, n (%)	7 (2.2)	5 (1.9)	2 (4.1)	0.3015
Horse, n (%)	7 (2.2)	5 (1.9)	2 (4.1)	0.3015
Small mammals^#^, n (%)	6 (1.9)	5 (1.9)	1 (2.0)	> 0.9999
Others (birds), n (%)	3 (1.0)	2 (0.8)	1 (2.0)	0.4010
Previous contact to animals (any)	313 (100)			
Cat, n (%)	280 (89.5)	239 (90.5)	41 (83.7)	0.2010
Dog, n (%)	285 (91.1)	242 (91.7)	43 (87.8)	0.4118
Horse, n (%)	226 (72.2)	**207 (78.4)**	**19 (38.8)**	**< 0.0001**
Small mammals^&^, n (%)	275 (87.9)	**238 (90.2)**	**37 (75.5)**	**0.0077**
Others (non-mammals)^§^, n (%)	210 (67.1)	175 (66.3)	35 (71.4)	0.5133
Farm animals^$^, n (%)	164 (52.4)	136 (51.5)	28 (57.1)	0.5344
Current pet ownership (any)	115 (36.7)	99 (37.5)	16 (32.7)	0.6288
Cat, n (%)	51 (16.3)	**49 (18.6)**	**2 (4.1)**	**0.0103**
Dog, n (%)	63 (20.1)	54 (20.5)	9 (18.4)	0.8475
Small mammals^&^, n (%)	28 (8.9)	26 (9.8)	2 (4.1)	0.2773
Others (non-mammals)^§^, n (%)	27 (8.6)	20 (7.6)	7 (14.3)	0.1606

*Females vs. males, Fisher’s exact test; **doctor’s diagnosis; ***self-reported symptoms; ^#^veterinary technicians, animal caretaker, farmer, biologist, veterinary technical assistant, animal healer, hoof orthopedist, equine ostheopath, butcher; ^&^rabbit, guinea pig, hamster, gerbil, mouse, rat, chinchilla, ferret, degus, marten, hedgehog; ^§^birds (incl. poultry), fish, reptiles; ^$^cattle, pig, goat, sheep. Bold = significantly different frequencies.

**Table 2.** Total and specific IgE antibodies.

		**Total (n = 313)**	**Females (n = 264)**	**Males (n = 49)**	p-value*
Total IgE > 100 kU/L	n (%)	83 (26.5%)	68 (25.8%)	15 (30.6%)	0.484
	IgE kU/L	218 (103 – 3,436)	220 (103 – 3,436)	168 (110 – 492)	
sx1 positives	n (%)	120 (38.4%)	**92 (34.8%)**	**28 (57.1%)**	**0.004**
	sIgE kU/L	8.64 (0.36 – 883)	8.63 (0.36 – 883)	9.80 (0.39 – 54.2)	
HDM positives	n (%)	76 (24.3%)	59 (22.3%)	17 (34.7%)	0.071
	sIgE kU/L	9.34 (0.36 – 518)	8.39 (0.36 – 518)	11.6 (0.40 – 20.4)	
Cat positives	n (%)	34 (10.9%)	29 (11.0%)	5 (10.2%)	>0.999
	sIgE kU/L	1.65 (0.36 – 244)	1.97 (0.36 – 244)	0.72 (0.58 – 7.52)	
Dog positives	n (%)	22 (7.0%)	18 (6.8%)	4 (8.2%)	0.760
	sIgE kU/L	0.98 (0.36 – 8.72)	0.98 (0.36 – 8.72)	0.98 (0.43 – 3.34)	
Horse positives	n (%)	16 (5.1%)	15 (5.7%)	1 (2.0%)	0.482
	sIgE kU/L	0.59 (0.36 – 7.73)	0.56 (0.36 – 7.73)	1.93	
Cattle positives	n (%)	8 (2.6%)	5 (1.9%)	3 (6.1%)	0.114
	sIgE kU/L	0.55 (0.36 – 1.79)	0.50 (0.36 – 1.20)	1.01 (0.45 – 1.06)	

IgE concentrations in kU/L are presented as median with range. *Females vs. males, Fisher’s exact test. sIgE = specific IgE; HDM = house dust mite. Bold = significantly different frequencies.

## References

[1] Linneberg A Gislum M Johansen N Husemoen LLN Jørgensen T Temporal trends of aeroallergen sensitization over twenty-five years. Clin Exp Allergy. 2007; 37: 1137–1142. 17651142 10.1111/j.1365-2222.2007.02760.x

[2] Warm K Lindberg A Lundbäck B Rönmark E Increase in sensitization to common airborne allergens among adults – two population-based studies 15 years apart. Allergy Asthma Clin Immunol. 2013; 9: 20. 23758681 10.1186/1710-1492-9-20 PMC3684537

[3] Kölli F Breyer M-K Hartl S Burghuber O Wouters EFM Sigsgaard T Pohl W Kohlböck G Breyer-Kohansal R Aero-Allergen Sensitization in the General Population: Longitudinal Analyses of the LEAD (Lung Heart Social Body) Study. J Asthma Allergy. 2022; 15: 461–473. 35431559 10.2147/JAA.S349614 PMC9012316

[4] Zahradnik E Raulf M Respiratory Allergens from Furred Mammals: Environmental and Occupational Exposure. Vet Sci. 2017; 4: 4. 29056697 10.3390/vetsci4030038 PMC5644656

[5] Bonini S Buonacucina A Occupational Hazards in Veterinarians: An Updating. J Veterinar Sci Technol. 2015; 07.

[6] Hoffmeyer F Beine A Lotz A Kleinmüller O Nöllenheidt C Zahradnik E Nienhaus A Raulf M Upper and lower respiratory airway complaints among female veterinary staff. Int Arch Occup Environ Health. 2022; 95: 665–675. 34669024 10.1007/s00420-021-01798-5 PMC8938376

[7] Fowler HN Holzbauer SM Smith KE Scheftel JM Survey of occupational hazards in Minnesota veterinary practices in 2012. J Am Vet Med Assoc. 2016; 248: 207–218. 26720089 10.2460/javma.248.2.207 PMC5710733

[8] Samadi S Wouters IM Heederik DJJ A review of bio-aerosol exposures and associated health effects in veterinary practice. Ann Agric Environ Med. 2013; 20: 206–221. 23772565

[9] Susitaival P Kirk JH Schenker MB Atopic symptoms among California veterinarians. Am J Ind Med. 2003; 44: 166–171. 12874849 10.1002/ajim.10253

[10] Samadi S Spithoven J Jamshidifard A-R Berends BR Lipman L Heederik DJJ Wouters IM Allergy among veterinary medicine students in The Netherlands. Occup Environ Med. 2012; 69: 48–55. 21632519 10.1136/oem.2010.064089

[11] Lehnert M Beine A Hoffmeyer F Taeger D Brüning T Raulf M Self-Reported Survey on Allergy Symptoms Among First-Year Students in Veterinary Medicine: A Preamble to the AllergoVet Cohort Study. Adv Exp Med Biol. 2020; 1279: 9–14. 32170668 10.1007/5584_2020_502

[12] Hoffmeyer F Beine A Lehnert M Berresheim H Taeger D van Kampen V Sander I Zahradnik E Brüning T Raulf M The Pattern of Sensitization Influences Exhaled and Nasal Nitric Oxide Levels in Young Adults. Adv Exp Med Biol. 2020; 1279: 15–26. 32193864 10.1007/5584_2020_509

[13] Heinzerling LM Burbach GJ Edenharter G Bachert C Bindslev-Jensen C Bonini S Bousquet J Bousquet-Rouanet L Bousquet PJ Bresciani M Bruno A Burney P Canonica GW Darsow U Demoly P Durham S Fokkens WJ Giavi S Gjomarkaj M Gramiccioni C GA(2)LEN skin test study I: GA(2)LEN harmonization of skin prick testing: novel sensitization patterns for inhalant allergens in Europe. Allergy. 2009; 64: 1498–1506. 19772515 10.1111/j.1398-9995.2009.02093.x

[14] Bousquet P-J Chinn S Janson C Kogevinas M Burney P Jarvis D Geographical variation in the prevalence of positive skin tests to environmental aeroallergens in the European Community Respiratory Health Survey I. Allergy. 2007; 62: 301–309. 17298348 10.1111/j.1398-9995.2006.01293.x

[15] Schmitz R Ellert U Kalcklösch M Dahm S Thamm M Patterns of sensitization to inhalant and food allergens - findings from the German Health Interview and Examination Survey for Children and Adolescents. Int Arch Allergy Immunol. 2013; 162: 263–270. 24022179 10.1159/000353344

[16] Asarnoj A Hamsten C Wadén K Lupinek C Andersson N Kull I Curin M Anto J Bousquet J Valenta R Wickman M van Hage M Sensitization to cat and dog allergen molecules in childhood and prediction of symptoms of cat and dog allergy in adolescence: A BAMSE/MeDALL study. J Allergy Clin Immunol. 2016; 137: 813–821. 26686472 10.1016/j.jaci.2015.09.052 PMC6597346

[17] Rönmark E Warm K Bjerg A Backman H Hedman L Lundbäck B High incidence and persistence of airborne allergen sensitization up to age 19 years. Allergy. 2017; 72: 723–730. 27659134 10.1111/all.13053

[18] Melén E Bergström A Kull I Almqvist C Andersson N Asarnoj A Borres MP Georgellis A Pershagen G Westman M van Hage M Ballardini N Male sex is strongly associated with IgE-sensitization to airborne but not food allergens: results up to age 24 years from the BAMSE birth cohort. Clin Transl Allergy. 2020; 10: 15. 32489587 10.1186/s13601-020-00319-w PMC7247167

[19] Haftenberger M Laußmann D Ellert U Kalcklösch M Langen U Schlaud M Schmitz R Thamm M Prävalenz von Sensibilisierungen gegen Inhalations- und Nahrungsmittelallergene : Ergebnisse der Studie zur Gesundheit Erwachsener in Deutschland (DEGS1). Bundesgesundheitsblatt Gesundheitsforschung Gesundheitsschutz. 2013; 56: 687–697. 23703487 10.1007/s00103-012-1658-1

[20] Hemmer W Sestak-Greinecker G Braunsteiner T Wantke F Wöhrl S Molecular sensitization patterns in animal allergy: Relationship with clinical relevance and pet ownership. Allergy. 2021; 76: 3687–3696. 33914361 10.1111/all.14885

[21] Bjerg A Winberg A Berthold M Mattsson L Borres MP Rönmark E A population-based study of animal component sensitization, asthma, and rhinitis in schoolchildren. Pediatr Allergy Immunol. 2015; 26: 557–563. 26059105 10.1111/pai.12422

[22] Konradsen JR Nordlund B Onell A Borres MP Grönlund H Hedlin G Severe childhood asthma and allergy to furry animals: refined assessment using molecular-based allergy diagnostics. Pediatr Allergy Immunol. 2014; 25: 187–192. 24460778 10.1111/pai.12198

[23] Roger A Lazo C Arias N Quirant B Albert N Gómez M Schayman W Using Component-Resolved Diagnosis to Characterize the Sensitization to Specific Cat and Dog Allergens in Patients with Allergic Respiratory Diseases in Catalonia, Spain. Int Arch Allergy Immunol. 2023; 184: 440–446. 36657403 10.1159/000528643 PMC10906471

[24] Liccardi G Asero R D’Amato M D’Amato G Role of sensitization to mammalian serum albumin in allergic disease. Curr Allergy Asthma Rep. 2011; 11: 421–426. 21809117 10.1007/s11882-011-0214-7

[25] Huang Z Zhu H Lin R Wu L An N Zheng P Sun B Serum Albumin as a Cross-Reactive Component in Furry Animals May Be Related to the Allergic Symptoms of Patients with Rhinitis. J Asthma Allergy. 2021; 14: 1231–1242. 34707374 10.2147/JAA.S334195 PMC8544268

[26] Apostolovic D Sánchez-Vidaurre S Waden K Curin M Grundström J Gafvelin G Cirkovic Velickovic T Grönlund H Thomas WR Valenta R Hamsten C van Hage M The cat lipocalin Fel d 7 and its cross-reactivity with the dog lipocalin Can f 1. Allergy. 2016; 71: 1490–1495. 27289080 10.1111/all.12955

[27] Hilger C Swiontek K Arumugam K Lehners C Hentges F Identification of a new major dog allergen highly cross-reactive with Fel d 4 in a population of cat- and dog-sensitized patients. J Allergy Clin Immunol. 2012; 129: 1149–1151. 22104604 10.1016/j.jaci.2011.10.017

[28] Nilsson OB Binnmyr J Zoltowska A Saarne T van Hage M Grönlund H Characterization of the dog lipocalin allergen Can f 6: the role in cross-reactivity with cat and horse. Allergy. 2012; 67: 751–757. 22515174 10.1111/j.1398-9995.2012.02826.x

[29] Dávila I Domínguez-Ortega J Navarro-Pulido A Alonso A Antolín-Amerigo D González-Mancebo E Martín-García C Núñez-Acevedo B Prior N Reche M Rosado A Ruiz-Hornillos J Sánchez MC Torrecillas M Consensus document on dog and cat allergy. Allergy. 2018; 73: 1206–1222. 29318625 10.1111/all.13391

[30] Burbach GJ Heinzerling LM Edenharter G Bachert C Bindslev-Jensen C Bonini S Bousquet J Bousquet-Rouanet L Bousquet PJ Bresciani M Bruno A Canonica GW Darsow U Demoly P Durham S Fokkens WJ Giavi S Gjomarkaj M Gramiccioni C Haahtela T GA(2)LEN skin test study II: clinical relevance of inhalant allergen sensitizations in Europe. Allergy. 2009; 64: 1507–1515. 19772516 10.1111/j.1398-9995.2009.02089.x

[31] Sánchez A Cardona R Munera M Calvo V Tejada-Giraldo M Sánchez J Nasal Provocation Test with Cat and Dog Extracts: Results according to Molecular Components. Pulm Med. 2020; 2020: 6365314. 32047667 10.1155/2020/6365314 PMC7001676

[32] Käck U Asarnoj A Grönlund H Borres MP van Hage M Lilja G Konradsen JR Molecular allergy diagnostics refine characterization of children sensitized to dog dander. J Allergy Clin Immunol. 2018; 142: 1113–1120. 29852259 10.1016/j.jaci.2018.05.012

[33] Simpson A Custovic A Pets and the development of allergic sensitization. Curr Allergy Asthma Rep. 2005; 5: 212–220. 15842959 10.1007/s11882-005-0040-x

[34] Chen C-M Tischer C Schnappinger M Heinrich J The role of cats and dogs in asthma and allergy--a systematic review. Int J Hyg Environ Health. 2010; 213: 1–31. 20053584 10.1016/j.ijheh.2009.12.003

[35] Lødrup Carlsen KC Roll S Carlsen K-H Mowinckel P Wijga AH Brunekreef B Torrent M Roberts G Arshad SH Kull I Krämer U von Berg A Eller E Høst A Kuehni C Spycher B Sunyer J Chen C-M Reich A Asarnoj A Does pet ownership in infancy lead to asthma or allergy at school age? Pooled analysis of individual participant data from 11 European birth cohorts. PLoS One. 2012; 7: e43214. 22952649 10.1371/journal.pone.0043214 PMC3430634

[36] Pinot de Moira A Strandberg-Larsen K Bishop T Pedersen M Avraam D Cadman T Calas L Casas M de Lauzon Guillain B Elhakeem A Esplugues A Estarlich M Foong RE Haakma S Harris JR Huang R-C Inskip H Lertxundi A Mensink-Bout SM Nader JLT Associations of early-life pet ownership with asthma and allergic sensitization: A meta-analysis of more than 77,000 children from the EU Child Cohort Network. J Allergy Clin Immunol. 2022; 150: 82–92. 35150722 10.1016/j.jaci.2022.01.023

[37] Bjerg A Ekerljung L Eriksson J Näslund J Sjölander S Rönmark E Dahl Å Holmberg K Wennergren G Torén K Borres MP Lötvall J Lundbäck B Increase in pollen sensitization in Swedish adults and protective effect of keeping animals in childhood. Clin Exp Allergy. 2016; 46: 1328–1336. 27159904 10.1111/cea.12757

[38] Taniguchi Y Kobayashi M Exposure to dogs and cats and risk of asthma: A retrospective study. PLoS One. 2023; 18: e0282184. 36888591 10.1371/journal.pone.0282184 PMC9994694

[39] Hesselmar B Hicke-Roberts A Lundell A-C Adlerberth I Rudin A Saalman R Wennergren G Wold AE Pet-keeping in early life reduces the risk of allergy in a dose-dependent fashion. PLoS One. 2018; 13: e0208472. 30566481 10.1371/journal.pone.0208472 PMC6300190

[40] Hesselmar B Aberg B Eriksson B Björkstén B Aberg N High-dose exposure to cat is associated with clinical tolerance – -a modified Th2 immune response? Clin Exp Allergy. 2003; 33: 1681–1685. 14656355 10.1111/j.1365-2222.2003.01821.x

[41] Heinrich J Gehring U Douwes J Koch A Fahlbusch B Bischof W Wichmann HE Pets and vermin are associated with high endotoxin levels in house dust. Clin Exp Allergy. 2001; 31: 1839–1845. 11737034 10.1046/j.1365-2222.2001.01220.x

[42] Fuertes E Standl M Markevych I Bischof W Heinrich J Is the association between pet ownership and indoor endotoxin levels confounded or modified by outdoor residential greenspace? Sci Total Environ. 2018; 625: 716–721. 29306159 10.1016/j.scitotenv.2017.12.333

[43] Schuijs MJ Willart MA Vergote K Gras D Deswarte K Ege MJ Madeira FB Beyaert R van Loo G Bracher F von Mutius E Chanez P Lambrecht BN Hammad H Farm dust and endotoxin protect against allergy through A20 induction in lung epithelial cells. Science. 2015; 349: 1106–1110. 26339029 10.1126/science.aac6623

[44] Gómez-Gallego C Forsgren M Selma-Royo M Nermes M Collado MC Salminen S Beasley S Isolauri E The Composition and Diversity of the Gut Microbiota in Children Is Modifiable by the Household Dogs: Impact of a Canine-Specific Probiotic. Microorganisms. 2021; 9: 9. 10.3390/microorganisms9030557 PMC8001081 33800493

[45] Song SJ Lauber C Costello EK Lozupone CA Humphrey G Berg-Lyons D Caporaso JG Knights D Clemente JC Nakielny S Gordon JI Fierer N Knight R Cohabiting family members share microbiota with one another and with their dogs. eLife. 2013; 2: e00458. 23599893 10.7554/eLife.00458 PMC3628085

[46] Tun HM Konya T Takaro TK Brook JR Chari R Field CJ Guttman DS Becker AB Mandhane PJ Turvey SE Subbarao P Sears MR Scott JA Kozyrskyj AL Exposure to household furry pets influences the gut microbiota of infant at 3-4 months following various birth scenarios. Microbiome. 2017; 5: 40. 28381231 10.1186/s40168-017-0254-x PMC5382463

[47] Rooks MG Garrett WS Gut microbiota, metabolites and host immunity. Nat Rev Immunol. 2016; 16: 341–352. 27231050 10.1038/nri.2016.42 PMC5541232

[48] Wlasiuk G Vercelli D The farm effect, or: when, what and how a farming environment protects from asthma and allergic disease. Curr Opin Allergy Clin Immunol. 2012; 12: 461–466. 22892709 10.1097/ACI.0b013e328357a3bc

[49] Campbell BE Lodge CJ Lowe AJ Burgess JA Matheson MC Dharmage SC Exposure to ‘farming’ and objective markers of atopy: a systematic review and meta-analysis. Clin Exp Allergy. 2015; 45: 744–757. 25270644 10.1111/cea.12429

[50] Eller E Roll S Chen C-M Herbarth O Wichmann H-E von Berg A Krämer U Mommers M Thijs C Wijga A Brunekreef B Fantini MP Bravi F Forastiere F Porta D Sunyer J Torrent M Høst A Halken S Lødrup Carlsen KC Meta-analysis of determinants for pet ownership in 12 European birth cohorts on asthma and allergies: a GA2LEN initiative. Allergy. 2008; 63: 1491–1498. 18721248 10.1111/j.1398-9995.2008.01790.x

